# CMIP Promotes Proliferation and Metastasis in Human Glioma

**DOI:** 10.1155/2017/5340160

**Published:** 2017-07-04

**Authors:** Bin Wang, Zheng-sheng Wu, Qiang Wu

**Affiliations:** ^1^Department of Oncology, The Second Affiliated Hospital of Anhui Medical University, Hefei, China; ^2^Department of Neurosurgery, The First Affiliated Hospital of Anhui Medical University, Hefei, Anhui, China; ^3^Department of Pathology, The Second Affiliated Hospital of Anhui Medical University, Hefei, China

## Abstract

Glioma is one of the most common primary malignant brain tumors and the outcomes are generally poor. The intrinsic mechanisms involved in glioma development and progression remain unclear. Further studies are urgent and necessary. In this study, we have proven that CMIP (C-Maf-inducing protein) promotes cell proliferation and metastasis in A172 cells through knockdown of CMIP and in U251 cells through overexpression of CMIP by using MTT assay, cell colony formation assay, cell migration assay, and cell invasion assay. Furthermore, we discovered that CMIP upregulates MDM2, which is involved in the promoting role of CMIP in human glioma cells. For clinical study, 99 glioma tissues and 59 normal tissues were analyzed. CMIP expression was higher in glioma tissues than in normal tissues. In glioma tissues, CMIP is found to correlate positively with tumor grade but no significant correlation is found with patients' age, gender, or Karnofsky performance score (KPS). Moreover, CMIP also correlates with low relapse-free survival (RFS) rate and overall survival (OS) rate in glioma patients. Therefore, CMIP is oncogenic and could be a potential target for human glioma diagnosis and therapy.

## 1. Introduction

Glioma is one of the most common primary malignant brain tumors which occur in both children and adults [1, 2]. Conventional treatments for glioma including surgical resection, radiation, and chemotherapy have shown limited impact [3, 4]. Even with optimal treatment, the average survival of glioma patients is no more than 1.5 years and the 5-year survival rate no more than 5% [4–6]. Recent studies focus on cellular and molecular mechanisms related to tumor initiation, development, and progression of human glioma, but the intrinsic mechanisms remain unclear. Further studies to improve the understanding of human glioma and to identify potential targets for therapy are critical.

CMIP (C-Maf-inducing protein) is expressed mainly in human brains and encodes an 86-kDa protein [7–9], which plays a role in T-cell signaling pathway and was firstly found to be abnormal in T-cells of minimal change nephritic syndrome (MCNS) patients [10, 11]. CMIP is an adaptor protein that contains two isoforms (a short protein and a long protein). The short protein isoform contributes to several biological pathways and the function of the long protein isoform is rarely known. Several reports have demonstrated that CMIP participates in human kidney diseases through regulating behaviors of podocytes [8, 12, 13]. CMIP is also reported to be associated with reading-related behaviors [14, 15], short-term memory, and language-related traits [7, 16]. However, thus far, there is no publication that documents the relation between CMIP and human tumor behaviors.

We studied the localization and expression of CMIP in human glioma cells A172 and U251 (these were chosen because they showed the highest and lowest levels of CMIP expression, resp.) and determined whether CMIP promotes both cell proliferation and metastasis by using MTT (3-(4,5-dimethyl-2-thiazolyl)-2,5-diphenyl-2-H-tetrazolium bromide) assay, cell colony formation assay, migration assay, and invasion assay in vitro. As reported previously, MDM2 was associated with tumor initiation and development of human glioma [17, 18]. Here we have shown that MDM2 is positively regulated by CMIP and may participate in the promoting role of CMIP in human glioma cells. Furthermore, we documented a much higher protein level of CMIP in human glioma tissues than that in normal tissues, and CMIP correlates positively with tumor grade in these glioma tissues. Patients with high expression of CMIP exhibited both lower relapse-free survival (RFS) and overall survival (OS) rates compared to patients with low expression of CMIP. Therefore, the oncogenicity of CMIP can be used as a potential target for diagnosis and therapy of human glioma.

## 2. Materials and Methods

### 2.1. Cell Lines and Cell Culture

Human glioma cells H4, A172, and U251 were obtained from the American Type Culture Collection (ATCC) (Rockville, MD). All of the cells were cultured in a humidified incubator at 37°C and 5% CO_2_ as recommended.

### 2.2. Plasmid and siRNA Transfection

The plasmid pIRESneo3 was obtained from Invitrogen. pIRESneo3 contains the internal ribosome entry site of the encephalomyocarditis virus and neomycin resistant region. Gene overexpression was done by G418 selection. Coding sequence of human CMIP was cloned into the plasmid pIRESneo3 (Invitrogen) and was used for CMIP overexpression. siRNA of CMIP obtained from GenePharma (Shanghai, China) was used for CMIP blocking. In this study, pIRESneo3-CMIP, pIRESneo3-Negative control, CMIP-siRNA, and negative control-siRNA were all transfected into cells by using Lip2000 (QIAGEN).

### 2.3. RT-Quantitative PCR (RT-qPCR)

RT-qPCR was used to determine the mRNA levels of CMIP in different human glioma cell lines, which was performed as described in previous studies using SYBR Premix Ex Taq Kit (Takara) [3]. GAPDH was used as a control. Primers for CMIP were the following: forward 5′-AAATTCCTGAGGCGCTG-3′; reverse 5′-CTTCAATTGCGCTGTAGGA-3′. Primers for GAPDH were the following: forward 5′-TGCACCACCAACTGCTTAGC-3′; reverse 5′-GGCATGGACTGTGGTCATGAG-3′.

### 2.4. Cell Oncogenicity Assays and Flow Cytometry Analysis

MTT assay and cell colony formation assay were performed to determine cell proliferation. Cell migration assay and invasion assay were performed to determine cell metastasis. They were all carried out as described previously [3, 19]. In MTT assay, cells were plated into 96-well plates with an original number of 2000 and tested using OD490 values after 1, 2, 3, and 4 days. In cell colony formation assay, cells were plated into 6-well plates with an original number of 5000 and tested after 1 week. For cell migration and invasion assay, cells were seeded and examined using 24-well transwell chambers (Corning, NY, USA) with or without matrigel coat. Cell apoptosis was examined by flow cytometry analysis using double-staining with Annexin V-FITC and PI in A172 cells.

### 2.5. Western Blot

In this study, protein level of CMIP (Proteintech 12851-1-AP1:1000) and MDM2 (Santa Cruz sc-53041:1000) in A172 and U251 cells were determined by Western blot analysis, as described in previous studies [3]. *α*-Tubulin (Proteintech 66031-1-Ig 1:5000) was used as a control. CMIP and MDM2 content of the cells were expressed in relation to the level of *α*-Tubulin.

### 2.6. Patients and Tissue Samples

99 paraffin-embedded surgical glioma tissue specimens and 59 normal tissue specimens were collected at the First Affiliated Hospital of Anhui Medical University (Hefei, Anhui, China) between 2009 and 2015. No other diseases were diagnosed and no special therapies were used in these patients before surgery. The clinicopathological parameters of patients with glioma were determined based on the World Health Organization grading systems. The patients with glioma were followed up for no less than 48 months. Informed consent was obtained from each patient before we performed this study. Our study plan has been approved by the institutional review board.

### 2.7. Immunohistochemistry (IHC)

Protein expression of CMIP in paraffin sections of human glioma tissues was examined using immunohistochemistry analysis. Similar to previous studies, immunohistochemistry analysis was carried out using two-step histostaining method (Maixin, Fuzhou, China) [3]. Briefly, the tissues were deparaffinized using exlene and rehydrated using graded series of ethanol solutions; antigen was retrieved using microwave oven heating method; endogenous peroxidase activity was blocked using 3% hydrogen peroxide; tissues on the sections were incubated with primary CMIP antibody (Proteintech 12851-1-AP 1:100) for 4 hours in 37°C; tissues were incubated with universal horseradish peroxidase-conjugated detection reagent (Maixin Biotech Co., Ltd.) for 20 min in 37°C; positive signals were detected using 3,3′-diaminobenzidine tetrahydrochloride (Maixin Biotech Co., Ltd.); tissues were counterstained using hematoxylin. An Olympus microscopy (Olympus America, Inc., Melville, NY) was used to evaluate the stained sections. Sections with 10% or more stained cells were designated as CMIP-positive, and sections with less than 10% stained cells were designated as CMIP-negative.

### 2.8. Statistical Analyses

All experiments were repeated at least three times. Unpaired two-tailed t-test was used for RT-qPCR, MTT assay, cell colony formation assay, cell migration assay, cell invasion assay, and flow cytometry analysis. For immunohistochemistry assay and clinicopathological parameters analysis, Pearson's chi-square test was used to analyze the significance of the differences. Kaplan-Meier curves were drafted to document the differences of patient relapse-free survival (RFS) and overall survival (OS) between high CMIP group and low CMIP group, and log-rank test was used to analyze the differences. The differences were considered significant when *P* < 0.05.

## 3. Results

### 3.1. CMIP Promotes Proliferation of Human Glioma Cells

**Figure 1:**
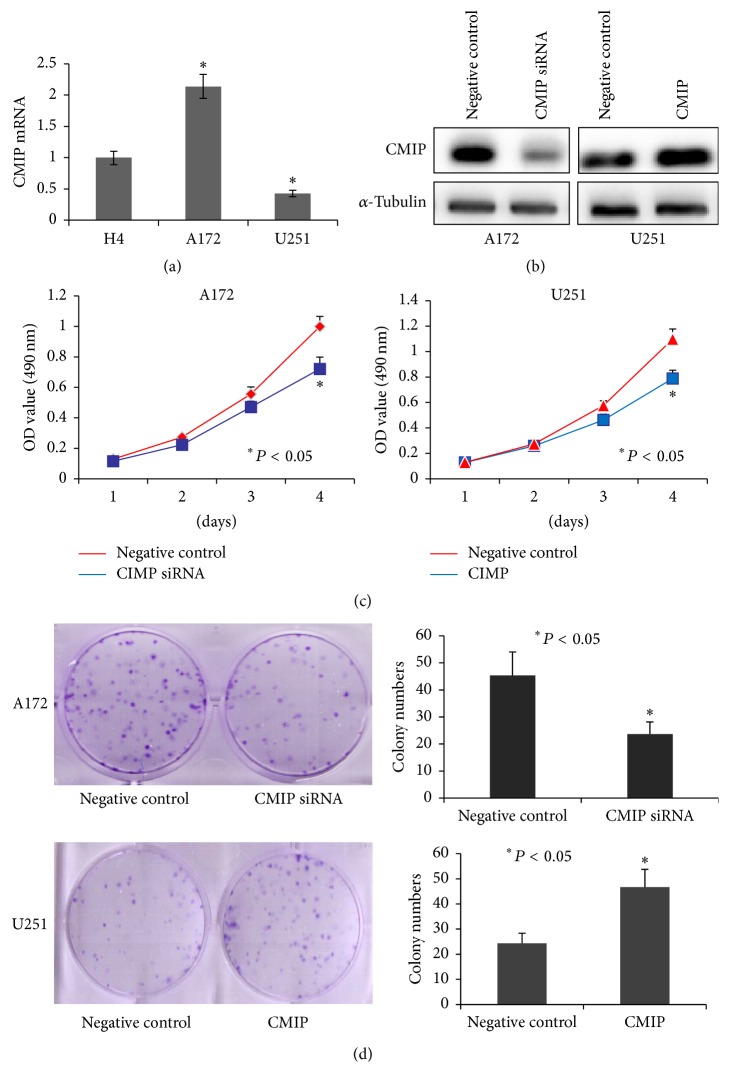
CMIP promotes proliferation of human glioma cells. (a) mRNA level of CMIP was examined in human glioma cells H4, A172, and U251 by using RT-qPCR. (b) Protein level of CMIP was tested after transfection with CMIP-siRNA or negative control siRNA in A172 cells and after transfection with pIRESneo3-CMIP or pIRESneo3-Negative control in U251 cells by Western blot. (c) MTT assay and (d) cell colony formation assay were carried out in A172 cells after transfection with CMIP-siRNA or negative control siRNA and in U251 cells after transfection with pIRESneo3-CMIP or pIRESneo3-Negative control, respectively. ^*∗*^*P* < 0.05.

To evaluate the base level of CMIP in different glioma cells, RT-qPCR was carried out in human glioma cells H4, A172, and U251. Among the three cell lines, mRNA level of CMIP was the highest in A172 cells and the lowest in U251 cells (Figure 1(a)). Therefore, we selected A172 to perform CMIP knockdown-related experiments and U251 for CMIP overexpressing-related experiments. Compared with negative control, CMIP-siRNA dramatically decreased the protein level of CMIP while pIRESneo3-CMIP dramatically increased the protein level of CMIP (Figure 1(b)). In A172 cells, cell viability decreased significantly over a period of 4 days after transfection with CMIP-siRNA compared with negative control siRNA (*P* < 0.05); meanwhile, cell viability increased significantly over a period of 4 days in U251 cells after transfection with pIRESneo3-CMIP compared with pIRESneo3-Negative control (*P* < 0.05) (Figure 1(c)). Concordantly, CMIP-siRNA significantly decreased cell colony formation of A172 cells (45 ± 10 versus 23 ± 5, *P* < 0.05) and pIRESneo3-CMIP significantly promoted cell colony formation of U251 cells (24 ± 4 versus 47 ± 7, *P* < 0.05) (Figure 1(d)). Moreover, flow cytometry analysis of A172 cells showed that the apoptotic cell population was 3.1 ± 0.3% in the negative control group and was 4.4 ± 0.3% in the CMIP-siRNA transfected group (*P* > 0.05) (see Figure  S1 in Supplementary Material available online at https://doi.org/10.1155/2017/5340160). As such, it can be deduced that CMIP promotes the proliferation of human glioma cells, but no significant change has been detected in the proportion of apoptotic cells.

### 3.2. CMIP Promotes Metastasis of Human Glioma Cells

**Figure 2:**
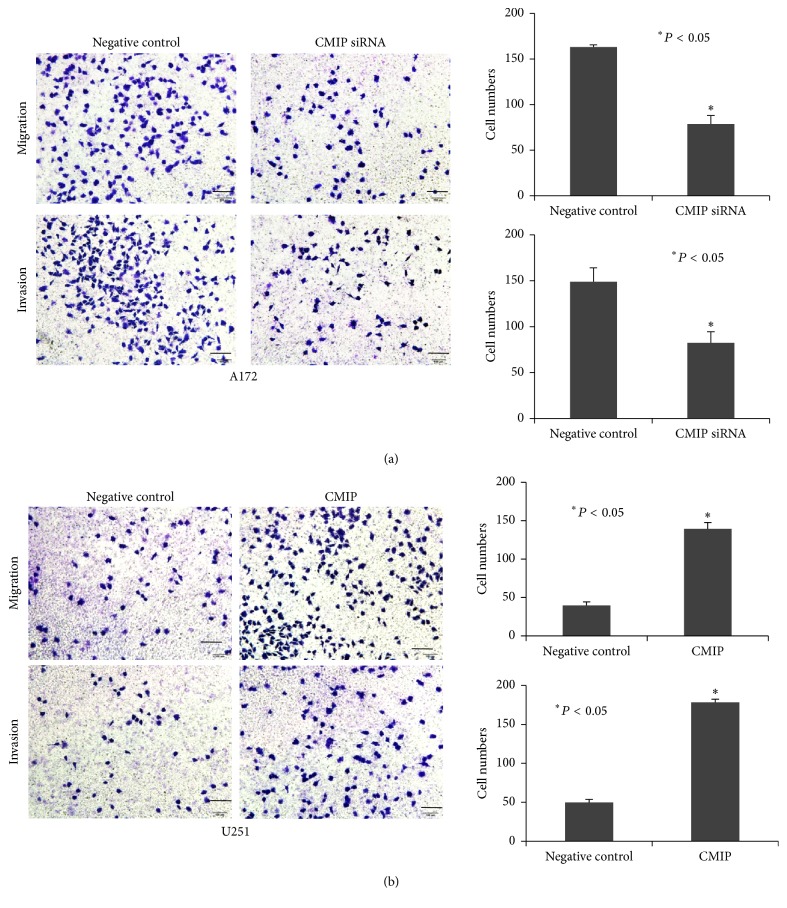
CMIP promotes metastasis of human glioma cells. A172 cells were transfected with CMIP-siRNA or negative control siRNA and U251 cells were transfected with pIRESneo3-CMIP or pIRESneo3-Negative control. (a) Migration assay and invasion assay were performed in A172 cells after transfection. (b) Migration assay and invasion assay were performed in U251 cells after transfection. ^*∗*^*P* < 0.05.

For further study, migration assay and invasion assay in human glioma cells were performed to examine the role of CMIP in cell metastasis. In A172 cells, both migration (171 ± 3 versus 78 ± 10, *P* < 0.05) and invasion (149 ± 15 versus 76 ± 13, *P* < 0.05) decreased significantly after transfection with CMIP-siRNA compared with control (Figure 2(a)). Concordantly, both migration (42 ± 5 versus 145 ± 8, *P* < 0.05) and invasion (48 ± 3 versus 176 ± 4, *P* < 0.05) increased significantly in U251 cells with overexpression of CMIP compared with control (Figure 2(b)). Therefore, CMIP also promotes metastasis of human glioma cells.

### 3.3. CMIP Regulates the Expression of MDM2

**Figure 3:**
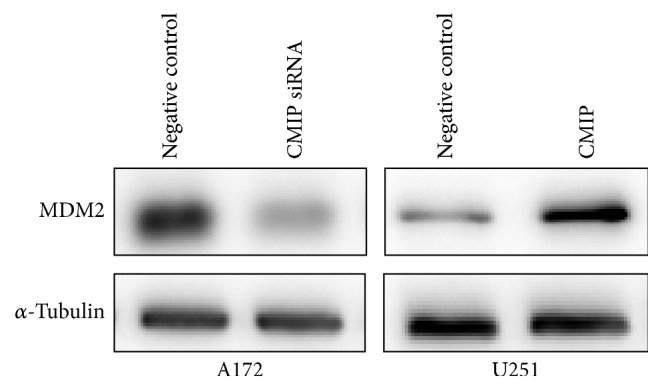
CMIP regulates the expression of MDM2. Protein level of MDM2 was tested after transfection with CMIP-siRNA or negative control siRNA in A172 cells and after transfection with pIRESneo3-CMIP or pIRESneo3-Negative control in U251 cells by Western blot. *α*-Tubulin was used as a control.

Next, we selected several candidate genes to identify the downstream mechanism of CMIP in human glioma cells. Among them, MDM2 decreased obviously after being transfected with CMIP-siRNA in A172 cells and increased significantly after transfection with pIRESneo3-CMIP in U251 cells (Figure 3). MDM2 has previously been reported as an oncogene [17, 18]. Therefore, MDM2 could be involved in the promoting role of CMIP in cell proliferation and metastasis of human glioma cells.

### 3.4. Association of CMIP Expression with Clinicopathological Parameters in Glioma Patients

**Figure 4:**
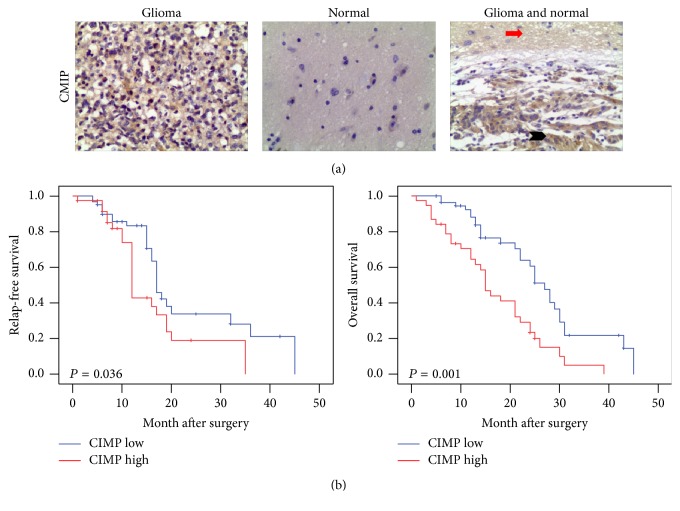
Association of CMIP expression with survival of glioma patients. (a) Expression of CMIP protein in glioma tissues and normal tissues was detected using immunohistochemistry. Representative 200x photographs were shown (red arrows: normal tissue; black arrows: glioma tissue). (b) Kaplan-Meier curves were made to show the difference in RFS and OS between high CMIP group and low CMIP group in glioma patients.

99 glioma tissue specimens and 59 normal tissue specimens were collected for clinical study. Protein levels of these tissues were detected using immunohistochemistry. As shown in Figure 4(a), positive signals of CMIP protein in the tumor cells were mainly located at the cytoplasm. CMIP expression was much higher in glioma tissues compared with normal tissues (Figure 4(a)). As shown in Table 1, in glioma tissues, 38 out of 99 cases (38.4%) were CMIP-positive and 61 out of 99 cases (61.6%) were CMIP-negative, whereas, in adjacent normal tissues, 12 out of 59 cases (20.3%) were CMIP-positive and 47 out of 59 cases (79.7%) were CMIP-negative. The difference of CMIP expression between glioma tissues and adjacent normal tissues was significant (*P* = 0.018).

In addition, we conducted association analysis between CMIP expression and clinicopathological parameters in the glioma tissue specimens. These clinicopathological parameters included patients' age, gender, KPS (Karnofsky performance score), and tumor grade. The percentage of tissues with high CMIP expression in high-grade gliomas (grades III-IV) (54.9%) was much higher than that in low-grade glioma (grades I-II) (20.8%) (*P* = 0.001). However, the differences between CMIP expression in relation to patients' age, gender, or KPS were not significant (all *P* > 0.1) (Table 2).

### 3.5. Association of CMIP Expression with Survival of Glioma Patients

Furthermore, we used the Kaplan-Meier curves to analyze the association of CMIP expression with RFS and OS rates in the 99 patients. All patients were followed up for more than 48 months. Compared with the low CMIP expression group, patients in the high CMIP expression group exhibited both lower RFS rate (*P* = 0.036) and lower OS rate (*P* = 0.001) (Figure 4(b)). Therefore, high CMIP expression is associated with poor prognosis in glioma patients.

## 4. Discussion

Herein we systematically studied the role of CMIP in human glioma both in vitro and in clinical tissues. Our study has confirmed CMIP as an oncogene in human glioma, and this is the first study to report the role of CMIP in human cancers. Both proliferation and metastasis dramatically decreased after blocking CMIP in glioma cell A172 with high expression of CMIP, whereas, in glioma cell U251 with low CMIP expression, both proliferation and metastasis dramatically increased after pIRESneo3-CMIP transfection. As for the downstream mechanism, MDM2 is found to be positively regulated by CMIP. Moreover, CMIP is expressed much higher in glioma tissues compared with normal tissues in our analysis of 99 glioma tissues and 59 normal tissues. CMIP is found to be positively associated with high tumor grade but not significantly associated with patients' age, gender, or KPS in these tissues. Furthermore, high expression of CMIP confers a worse prognosis in glioma patients, including lower RFS and OS.

High-grade glioma remains a severe problem because of its highly invasive and diffusive infiltrative nature. The optimal treatment for patients with glioma is surgical resection with chemotherapy, radiotherapy, or other adjuvant therapies. But the outcomes are typically poor, and the average survival of glioma patients is less than 18 months [1, 3, 20]. Studies on potential targets for molecular therapy of human glioma are critical. Previous publications have reported many miRNAs (microRNAs), oncogenes, and tumor suppressing genes involved in the proliferation and metastasis of human glioma. These miRNAs include miR-491, miR-433-3p, miR-221, and miR-16 [21–24]. The F-box protein FBXL18 promotes glioma progression by promoting K63-linked ubiquitination of Akt [25]. CXCL5 promotes the proliferation and migration of glioma cells [26]. On the other hand, KIAA0247 has been reported to play a tumor suppressing role in proliferation, angiogenesis, and promoting apoptosis of human glioma [27]. SOX7 is associated with the suppression of human glioma [28]. In this study, we confirmed that CMIP promotes both proliferation and metastasis of human glioma and demonstrated that CMIP is positively correlated with tumor grade and poor RFS and OS in glioma patients. We have therefore found a new potential target for glioma therapy.

As reported previously, MDM2 is associated with tumor initiation and development of human glioma. MDM2 promotes tumor proliferation and metastasis by downregulating tumor suppressing genes including p53 [17, 29]. Yan et al. have demonstrated that inhibition of MDM2 inhibits proliferation and motility of glioma cells [18]. It has also been reported that activating the MDM2-TP53 pathway increases cell apoptosis of glioma cells [30]. Herein, we verified that MDM2 is upregulated by CMIP. While CMIP acts as a tumor promoter in glioma, MDM2 is also reported to be an oncogene. MDM2 has also been reported to play tumor promoting role in human breast cancer, lung cancer, and colon cancer [31–33]. Therefore, it can be concluded that MDM2 contributes to the oncogenic role of CMIP in human glioma.

In conclusion, this study is the first to examine the oncogenic role of CMIP in human glioma. Systematical experiments including in vitro cell experiments and clinical studies were performed. High level of CMIP is associated with the more malignant nature of glioma cells and bad prognosis of patients with glioma. Our data suggested that CMIP could be used as a new potential therapeutic target for human glioma.

## Supplementary Material

Figure  S1. Flow cytometry analysis in A172 cells.

## Figures and Tables

**Table 1:** Expression of CMIP in glioma and adjacent normal tissues.

Group	*n*	CMIP expression	
		Negative, *n* (%)	Positive, *n* (%)
Tumor	99	61 (61.6)	38 (38.4)^*∗*^
Normal	59	47 (79.7)	12 (20.3)

*Note*. ^*∗*^*P* = 0.018.

**Table 2:** Association of CMIP expression with clinicopathological parameters from glioma patients.

Parameter	*n*	CMIP expression (*n* (%))		*P*
		Low	High	
Age (years)				
<50	62	38 (61.3)	24 (38.7)	0.931
≥50	37	23 (62.2)	14 (37.8)	
Gender				
Female	41	29 (70.7)	12 (29.3)	0.117
Male	58	32 (55.2)	26 (44.8)	
KPS				
<80	35	20 (57.1)	15 (42.9)	0.499
≥80	64	41 (64.1)	23 (35.9)	
Grade				
I-II	48	38 (79.2)	10 (20.8)	0.001
III-IV	51	23 (45.1)	28 (54.9)	

*Note*. KPS (Karnofsky performance score).

## References

[1] Chen S., Wang Y., Ni C., Meng G., Sheng X. (2016). HLF/miR-132/TTK axis regulates cell proliferation, metastasis and radiosensitivity of glioma cells. *Biomedicine and Pharmacotherapy*.

[2] Luo G., Luo W., Sun X. (2016). MicroRNA21 promotes migration and invasion of glioma cells via activation of Sox2 and betacatenin signaling. *Molecular Medicine Reports*.

[3] Jiang T., Zhao B., Li X., Wan J. (2016). ARPP-19 promotes proliferation and metastasis of human glioma. *NeuroReport*.

[4] Qi L., Yu H., Zhang Y. (2016). IL-10 secreted by M2 macrophage promoted tumorigenesis through interaction with JAK2 in glioma. *Oncotarget*.

[5] Rutka J. T., Taylor M., Mainprize T. (2000). Molecular biology and neurosurgery in the third millennium. *Neurosurgery*.

[6] Li J., Cai J., Zhao S. (2016). GANT61, a GLI inhibitor, sensitizes glioma cells to the temozolomide treatment. *Journal of Experimental and Clinical Cancer Research*.

[7] Newbury D. F., Winchester L., Addis L. (2009). CMIP and ATP2C2 modulate phonological short-term memory in language impairment. *American Journal of Human Genetics*.

[8] Sendeyo K., Audard V., Zhang S.-Y. (2013). Upregulation of c-mip is closely related to podocyte dysfunction in membranous nephropathy. *Kidney International*.

[9] Sahali D., Pawlak A., Valanciute A. (2002). A novel approach to investigation of the pathogenesis of active minimal-change nephrotic syndrome using subtracted cDNA library screening. *Journal of the American Society of Nephrology*.

[10] Liu Y., Su L., Lin Q., Han Y., You P., Fan Q. (2015). Induction of C-Mip by IL-17 plays an important role in adriamycin-induced podocyte damage. *Cellular Physiology and Biochemistry*.

[11] Grimbert P., Valanciute A., Audard V. (2003). Truncation of C-mip (Tc-mip), a new proximal signaling protein, induces c-maf Th2 transcription factor and cytoskeleton reorganization. *Journal of Experimental Medicine*.

[12] Moktefi A., Zhang S.-Y., Vachin P. (2016). Repression of CMIP transcription by WT1 is relevant to podocyte health. *Kidney International*.

[13] Ory V., Fan Q., Hamdaoui N. (2012). C-mip down-regulates NF-*κ*B activity and promotes apoptosis in podocytes. *American Journal of Pathology*.

[14] Kato M., Okanoya K., Koike T. (2014). Human speech- and reading-related genes display partially overlapping expression patterns in the marmoset brain. *Brain and Language*.

[15] Scerri T. S., Morris A. P., Buckingham L.-L. (2011). DCDC2, KIAA0319 and CMIP are associated with reading-related traits. *Biological Psychiatry*.

[16] Van der Aa N., Vandeweyer G., Reyniers E. (2012). Haploinsufficiency of CMIP in a girl with autism spectrum disorder and developmental delay due to a de novo deletion on chromosome 16q23.2. *Autism Research*.

[17] Wen C., Renliang W., Huiling C., Jifa G., Xu W., Qiwei R. (2005). P53 Gene mutation and expression of MDM2, P53, P16 protein and their relationship in human glioma. *Journal of Huazhong University of Science and Technology [Medical Sciences]*.

[18] Yan Y., Peng Y., Ou Y., Jiang Y. (2016). MicroRNA-610 is downregulated in glioma cells, and inhibits proliferation and motility by directly targeting MDM2. *Molecular Medicine Reports*.

[19] Ding Z.-Y., Jin G.-N., Wang W. (2014). Reduced expression of transcriptional intermediary factor 1 gamma promotes metastasis and indicates poor prognosis of hepatocellular carcinoma. *Hepatology*.

[20] Stupp R., Mason W. P., van den Bent M. J. (2005). Radiotherapy plus concomitant and adjuvant temozolomide for glioblastoma. *The New England Journal of Medicine*.

[21] Qi Z., Cai S., Cai J. (2016). miR-491 regulates glioma cells proliferation by targeting TRIM28 in vitro. *BMC Neurology*.

[22] Sun S., Wang X., Xu X. (2016). MiR-433-3p suppresses cell growth and enhances chemosensitivity by targeting CREB in human glioma. *Oncotarget*.

[23] Yang J.-K., Yang J.-P., Tong J. (2016). Exosomal miR-221 targets DNM3 to induce tumor progression and temozolomide resistance in glioma. *Journal of Neuro-Oncology*.

[24] Zhou Y., Liu Y., Hu C., Jiang Y. (2016). MicroRNA-16 inhibits the proliferation, migration and invasion of glioma cells by targeting Sal-like protein 4. *International Journal of Molecular Medicine*.

[25] Zhang J., Yang Z., Ou J., Xia X., Zhi F., Cui J. (2016). The F-box protein FBXL18 promotes glioma progression by promoting K63-linked ubiquitination of Akt. *FEBS Letters*.

[26] Dai Z., Wu J., Chen F. (2016). CXCL5 promotes the proliferation and migration of glioma cells in autocrine- and paracrine-dependent manners. *Oncology Reports*.

[27] Tan Y., Huang N., Zhang X. (2016). KIAA0247 suppresses the proliferation, angiogenesis and promote apoptosis of human glioma through inactivation of the AKT and Stat3 signaling pathway. *Oncotarget*.

[28] Zhao T., Yang H., Tian Y. (2016). SOX7 is associated with the suppression of human glioma by HMG-box dependent regulation of Wnt/*β*-catenin signaling. *Cancer Letters*.

[29] Danilovskyi S. V., Minchenko D. O., Moliavko O. S., Kovalevska O. V., Karbovskyi L. L., Minchenko O. H. (2014). ERN1 knockdown modifies the hypoxic re gulation of TP53, MDM2, USP7 and PERP gene expre ssions in U87 glioma cells. *Ukrainian Biochemical Journal*.

[30] Zhanfeng N., Chengquan W., Hechun X. (2016). Period2 downregulation inhibits glioma cell apoptosis by activating the MDM2-TP53 pathway. *Oncotarget*.

[31] Xu J., Han M., Shen J. (2016). 2-Methoxy-5((3,4,5-trimethosyphenyl)seleninyl) phenol inhibits MDM2 and induces apoptosis in breast cancer cells through a p53-independent pathway. *Cancer Letters*.

[32] Hai J., Sakashita S., Allo G. (2015). Inhibiting MDM2-p53 interaction suppresses tumor growth in patient-derived non-small cell lung cancer xenograft models. *Journal of Thoracic Oncology*.

[33] Rigatti M. J., Verma R., Belinsky G. S., Rosenberg D. W., Giardina C. (2012). Pharmacological inhibition of Mdm2 triggers growth arrest and promotes DNA breakage in mouse colon tumors and human colon cancer cells. *Molecular Carcinogenesis*.

