# Supplementary figures and images for: CMIP Promotes Proliferation and Metastasis in Human Glioma
Source: Biomed Res Int. 2017 Jul 4;2017:5340160. doi: 10.1155/2017/5340160 (PMC5514325; doi:10.1155/2017/5340160)

Fig. S1

A172

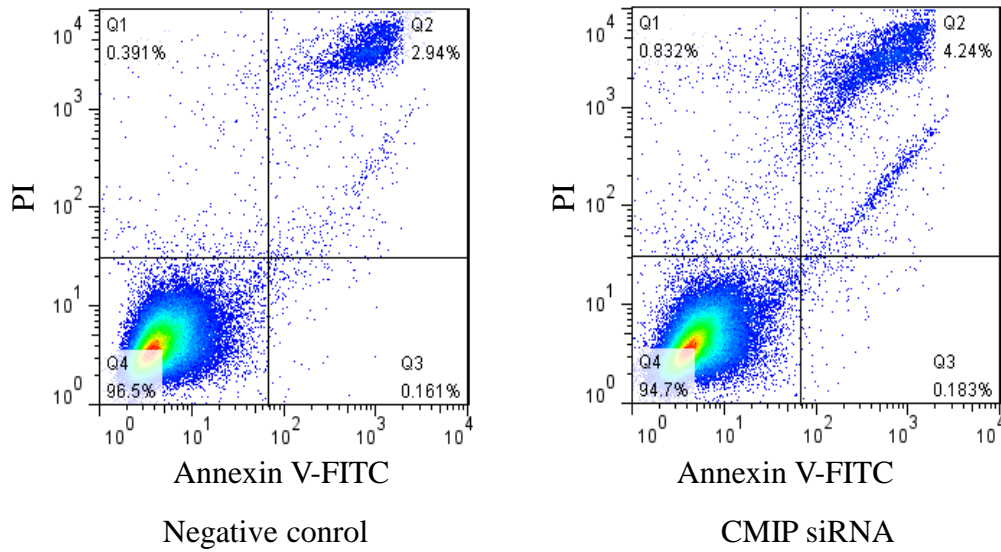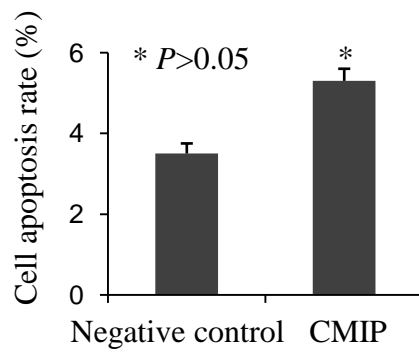

Supplement: Supplementary file 1 — Figure S1. Flow cytometry analysis in A172 cells. [file 5340160.f1.pdf]
